# Attentional modulation of beta-power aligns with the timing of behaviorally relevant rhythmic sounds

**DOI:** 10.1093/cercor/bhac179

**Published:** 2022-05-27

**Authors:** Maja D Foldal, Sabine Leske, Alejandro O Blenkmann, Tor Endestad, Anne-Kristin Solbakk

**Affiliations:** Department of Psychology, University of Oslo, Forskningsveien 3A, 0373 Oslo, Norway; RITMO Centre for Interdisciplinary Studies in Rhythm, Time and Motion, University of Oslo, Forskningsveien 3A, 0373 Oslo, Norway; RITMO Centre for Interdisciplinary Studies in Rhythm, Time and Motion, University of Oslo, Forskningsveien 3A, 0373 Oslo, Norway; Department of Musicology, University of Oslo, Sem Sælands vei 2, 0371 Oslo, Norway; Department of Psychology, University of Oslo, Forskningsveien 3A, 0373 Oslo, Norway; RITMO Centre for Interdisciplinary Studies in Rhythm, Time and Motion, University of Oslo, Forskningsveien 3A, 0373 Oslo, Norway; Department of Psychology, University of Oslo, Forskningsveien 3A, 0373 Oslo, Norway; RITMO Centre for Interdisciplinary Studies in Rhythm, Time and Motion, University of Oslo, Forskningsveien 3A, 0373 Oslo, Norway; Department of Neuropsychology, Helgeland Hospital, Skjervengan 17, 8657 Mosjøen, Norway; Department of Psychology, University of Oslo, Forskningsveien 3A, 0373 Oslo, Norway; RITMO Centre for Interdisciplinary Studies in Rhythm, Time and Motion, University of Oslo, Forskningsveien 3A, 0373 Oslo, Norway; Department of Neuropsychology, Helgeland Hospital, Skjervengan 17, 8657 Mosjøen, Norway; Department of Neurosurgery, Oslo University Hospital, Sognsvannsveien 20, 0372 Oslo, Norway

**Keywords:** electroencephalography, alpha-beta oscillations, auditory selective attention, lateralization, temporal prediction

## Abstract

It is largely unknown how attention adapts to the timing of acoustic stimuli. To address this, we investigated how hemispheric lateralization of alpha (7–13 Hz) and beta (14–24 Hz) oscillations, reflecting voluntary allocation of auditory spatial attention, is influenced by tempo and predictability of sounds. We recorded electroencephalography while healthy adults listened to rhythmic sound streams with different tempos that were presented dichotically to separate ears, thus permitting manipulation of spatial–temporal attention. Participants responded to stimulus-onset-asynchrony (SOA) deviants (−90 ms) for given tones in the attended rhythm. Rhythm predictability was controlled via the probability of SOA deviants per block. First, the results revealed hemispheric lateralization of beta-power according to attention direction, reflected as ipsilateral enhancement and contralateral suppression, which was amplified in high- relative to low-predictability conditions. Second, fluctuations in the time-resolved beta-lateralization aligned more strongly with the attended than the unattended tempo. Finally, a trend-level association was found between the degree of beta-lateralization and improved ability to distinguish between SOA-deviants in the attended versus unattended ear. Differently from previous studies, we presented continuous rhythms in which task-relevant and irrelevant stimuli had different tempo, thereby demonstrating that temporal alignment of beta-lateralization with attended sounds reflects top-down attention to sound timing.

## Introduction

Attention plays a crucial role in the goal-directed selection of relevant information in noisy environments. As the sensory environment is highly dynamic (i.e. moving objects, speech, and sounds), flexible allocation of attentional resources according to the timing of goal-relevant information is required (Lakatos et al. 2013; Nobre and van Ede 2018). In the literature, this is generally referred to as *temporal attention* (for reviews, see Correa et al. 2006; Morillon and Schroeder 2015; Nobre and van Ede 2018). Current knowledge of how attentional selection is implemented when sensory inputs have varying temporal structures, is sparse.

Top-down attention can be defined as a mechanism that reduces computational load by prioritizing aspects of the sensory environment most relevant to the perceiver’s current goals (Summerfield and Egner 2009; Frey et al. 2015). In neural terms, this is reflected as enhanced processing in functionally relevant cortical areas, as well as suppressed processing in irrelevant cortical areas (Foxe and Snyder 2011; Klimesch 2012). It has been proposed that this relative enhancement and suppression is achieved through modulation of ongoing oscillatory activity in the alpha (7–13 Hz) and beta (14–24 Hz) bands, also shown to be inversely related to cortical excitability (Jensen and Mazaheri 2010; Wöstmann et al. 2018; Griffiths et al. 2019). In studies in which spatial relevance (i.e. left vs. right) has been manipulated, a relative suppression of oscillatory power in the alpha- and beta-bands is typically found contralateral to the stimulated side (for a review, see Frey et al. 2015). This effect is apparent in studies manipulating the spatial relevance of stimuli within several sensory modalities, such as audition (Frey et al. 2014; Wöstmann et al. 2016; Deng et al. 2020), vision (Wildegger et al. 2017; Heideman et al. 2018a), as well as tactile sensation (van Ede et al. 2011; van Ede et al. 2014). Hence, it appears to reflect a general mechanism of attentional selection, independent of sensory modality.

Although the alpha-band has been mainly targeted in the study of top-down attentional selection and lateralized oscillatory power, the effects are not always confined to this band, but also involve the beta-band (i.e. Weisz et al. 2014). Moreover, modulation of lateralized power related to *temporal* anticipation has been reported to be more prominent for the beta- than the alpha-band (van Ede et al. 2011; Heideman et al. 2018b). van Ede et al. (2011) argued that while the alpha-band has been shown to be involved in spatial- as well as stimulus feature-specific anticipatory attention, attentional modulation of beta-band oscillations seems to reflect a mechanism of temporal orienting. This is in line with studies suggesting a specific role of beta-band oscillations in timing-related aspects of sensory processing (Fujioka et al. 2012; Arnal et al. 2015; Meijer et al. 2016; Morillon and Baillet 2017). Processing of temporality is assumed to rely on cortical action circuits involving motor and parietal regions (for a review, see Coull and Nobre 2008), and source localization of changes in beta-power due to timing-related processing has suggested involvement of sensorimotor areas (Fujioka et al. 2012; Arnal et al. 2015; Morillon and Baillet 2017). Furthermore, beta-oscillations have been linked to endogenous top-down controlled processing, although their precise functional role during temporal attention is not clear (for a review, see Spitzer and Haegens 2017). If previous studies are correct that the beta-band is predominantly involved in *temporal* aspects of attention, one might expect to see modulation primarily in this frequency band if a task involves decisions regarding stimulus-timing only (i.e. detecting time-deviances). So far, this has not been investigated, as tasks used in previous studies mainly involved decisions regarding other features of the presented stimuli (i.e. van Ede et al. 2011; Wöstmann et al. 2016; Heideman et al. 2018a).

It is also argued that modulation of beta-oscillations during temporal orienting could result from the top-down prediction of temporal structure (Arnal and Giraud 2012). Although earlier research consistently reported top-down influences (i.e. learning and memory) on perception (Engel et al. 2001; Gilbert and Sigman 2007), the organization of predictive processing in the human brain is not yet well documented. An account of how predictions may be organized in the human brain is given by the “predictive coding” framework (Friston 2005). One assumption of the theory is that the brain models the future sensory environment in a probabilistic manner, where predictions are shaped through statistical rules, specifically Bayesian inference (Aitchison and Lengyel 2017). Not only does the brain make predictions regarding “what” is expected, but also “when” it is expected (for a review, see Nobre and van Ede 2018). van Ede et al. (2011) showed a clear time-varying modulation of beta-power lateralization based on the expected temporal structure of stimuli. However, the experimental paradigm used in that study did not permit isolating effects related to sensory prediction from attention effects, as temporal expectancy (i.e. stimulus interval probability) was used to manipulate temporal orienting (attention). Interestingly, a recent study reported stronger attentional modulation of oscillatory power in the beta range for learned compared to novel visual-motor temporal sequences (Heideman et al. 2018b). Whether modulations of beta-band activity during learning of temporal sequences in fact represent learning probabilities of the specific intervals, i.e. through Bayesian inference, remains an open question.

Finally, the investigation of attentional modulation of alpha- and beta-activity has mainly involved experiments where the interval between a cue and a target stimulus is of main interest (i.e. Frey et al. 2014; Deng et al. 2020). These experiments do not represent well the complex temporal dynamics found in natural environments. Wöstmann et al. (2016) argued that the spatial allocation of selective attention might not be sufficient for selection of dynamic sensory input. Besides, our sensory environment often entails transient sounds that are irrelevant to the task at hand. Wöstmann et al. (2016) claimed that a neural mechanism of attention needs to be dynamically modulated over time. Interestingly, they found that lateralization of alpha-power synchronized with the presentation rate (i.e. tempo) of stimuli when participants had to report spoken digits from one ear, while at the same time ignoring spoken digits presented simultaneously to the opposite ear. Supporting the view of dynamic attention modulation, a human intracranial electroencephalography (EEG) study reported that the frontoparietal attention network indeed operates through rhythmic perceptual sampling (Helfrich et al. 2018). A potential limitation of the study by Wöstmann et al. (2016), where the relevant and irrelevant auditory input streams were presented in parallel, is that the two auditory streams had identical temporal structures and onsets (i.e. were synchronized). This is often not the case for auditory stimuli in our natural surroundings. The dynamics of selective attention when attended and distracting stimulus streams presented in parallel not only compete in terms of spatial location but also temporal structure (e.g. tempo), remain to be investigated.

In the current study, we independently manipulated spatial–temporal attention and temporal (rhythm) predictability in an experimental task involving judgments of rhythm timing, to investigate modulations of alpha- and beta-oscillatory activity. Participants were asked to attend to a stream of tones presented to one ear while ignoring another stream of tones presented simultaneously to the opposite ear. While the tones were identical between ears in terms of pitch, duration, and loudness, the two tone streams were presented with different tempos. This permitted manipulation of spatial- as well as temporal attention. Although different types of temporal structures guide temporal attention (for a review, see Nobre and van Ede 2018), here, temporal attention refers to how attention allocation adjusts to the *tempo* of behaviorally relevant isochronous stimuli. Combined, tones from the two ears comprised rhythmic sequences that were repeated throughout the experimental blocks. While it should be noted that these sequences were relatively short (2.4 s), the repetition of the sequences throughout each block comprised a continuous rhythm. The task involved detecting and responding to stimulus-onset asynchrony (SOA) deviances (−90 ms) in the rhythmic stimuli within the specified ear. The SOA deviants were presented rarely (i.e. high rhythm predictability) or frequently (i.e. low rhythm predictability) in separate experimental blocks.

The current study had three main aims: (1) We investigated how oscillatory power in the alpha-beta range is influenced by selective attention in a task that primarily involves decisions regarding stimulus timing, as all other stimulus features (i.e. pitch and duration) were constant. We expected to see lateralization of oscillatory power in the alpha-beta (7–24 Hz) range according to the spatial direction of attention (i.e. right vs. left ear). The functional role of beta-oscillations remains debated (Spitzer and Haegens 2017). However, we hypothesized stronger modulation of power in the beta-band (14–24 Hz) than in the alpha-band (7–13 Hz) given propositions that modulated beta-band activity during top-down attentional selection reflects timing-related processing (van Ede et al. 2011; Heideman et al. 2018b). (2) We also assessed whether attentional modulation is influenced by rhythm predictability. We expected a stronger impact of attention in the “high predictability” (i.e. few deviants) compared to the “low predictability” (i.e. many deviants) contexts provided that modulations of beta-oscillations during temporal orienting reflect predictive processing. (3) Finally, we addressed how selective attention adapts to varying temporal structures in auditory stimuli (i.e. slow vs. fast tempo). Given that attention selectively adapts to the temporal dynamics in behaviorally relevant stimuli, we expected attention modulation effects to align more strongly with the temporal structure of tones in the attended ear than to tones in the unattended ear. More specifically, when tones in the attended ear were presented with a fast tempo, we expected attention modulation to align more strongly with the faster compared to the slower tempo, and vice versa.

## Materials and methods

The current paper reports the results of a reanalysis of data from a previously published study (Foldal et al. 2020) in which we examined effects of rhythm predictability on auditory evoked potentials to attended and unattended sounds.

### Participants

A sample of 34 healthy adults was recruited for the study. Two participants were excluded as it was evident during recording that they did not follow instructions properly when performing the Dichotic Listening experimental task. Two participants were additionally excluded due to being considered outliers with respect to the electrophysiological data (see “Statistical analysis” below for details). This resulted in a sample of 30 subjects (16 females; 26 right-handed; mean age, 24.0 years; range, 19–35 years; SD, 4.2 years). All reported having accomplished high-school level education, and 27 were currently students at an institution for higher-level education or having a university or college degree.

All reported normal hearing, no neurological problems, and no cognitive difficulties. Participants also reported not currently receiving any psychiatric treatment, including no medication for mental illness. None were professional musicians (performing artists, music teachers, or conservatory students). Participants gave written informed consent before participation. The study was approved by the Department of Psychology’s internal research ethics committee (University of Oslo) and was conducted in agreement with the Declaration of Helsinki.

Note that it became clear throughout statistical analysis that the critical neural measure of attention modulation predicted the amount of behavioral errors in the experiment. Accordingly, another four participants were excluded (*n* = 26) for the remaining analyses, including analyses of the temporal progression of attention modulation effects, as well as investigation of links between attention modulation effects and behavioral performance.

### Stimuli and experimental design

**Fig. 1:**
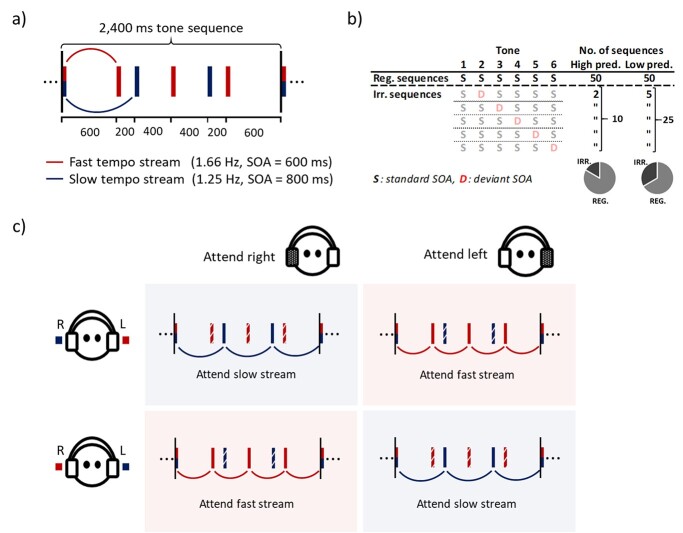
Illustration of the stimuli and experimental design. (a) The temporal structure of the 6-tone rhythm sequences (i.e. fast and slow stream combined), where all tones were identical in terms of pitch, duration, and loudness. Total duration of sequences (above) and SOA between tones (below) are indicated in milliseconds (ms). Colors indicate how the rhythm was dichotically presented, as tones of blue color (“slow” tempo = 1.25 Hz) were presented to one ear and tones of red color (“fast” tempo = 1.66 Hz) to the opposite ear (counterbalanced). (b) Schematic illustration of the number of regular (reg.) and irregular (irr.) rhythm sequences per high- and low-predictability (pred.) blocks. All possible irregular sequences are illustrated in terms of when the deviant (D) SOA was presented, the number of each irregular sequence per high- and low-predictability blocks, as well as the total number of irregular sequences per high- and low-predictability blocks. Irregular sequences are shaded as these were not analyzed. The gray circles indicate the proportion of regular and irregular sequences in the high- and low-predictability blocks. Note the discrepancy between the number of regular sequences presented in the experiment and the number used for analysis due to exclusion of sequences preceded by an irregular sequence (due to potential motor confound). (c) Counterbalancing within each participant; attended ear (top panels) and dichotic configuration (left panels). Sequences occurred in either “high”- or “low”-predictability blocks. This resulted in 8 different experimental blocks. Neural processing of the rhythm sequence was analyzed in terms of the spatial direction of attention (left ear vs. right ear) as well as predictability (high vs. low). Finally, neural processing of the rhythm sequence was analyzed in terms of attended tempo (solid blue vs. red lines = attend slow vs. attend fast), which varied according to the combination of dichotic configuration (left) and direction of attention (top). The broken blue or red lines represent the unattended tone stream of the rhythm sequence. Filled (gray) headphones indicate the attended ear.

Isochronous streams of tones were presented to each ear, in which all tones from either stream were identical in terms of duration (50 ms, 7 ms rising and falling periods), pitch (3 harmonics of a 220 Hz fundamental frequency), and loudness. The tones were presented at the same loudness level across conditions and participants. Importantly, the two streams had different tempos. One stream had a SOA of 800 ms (1.25 Hz presentation rate, “slow tempo”) and the other stream a SOA of 600 ms (1.66 Hz presentation rate, “fast tempo”). Combined, the two streams formed rhythmic sequences of 6 tones (total duration of 2.4 s) comprising a 3- versus 4-beat meter. This involved the presentation of 3 tones to one ear (“slow tempo”) and 4 tones to the other ear (“fast tempo”), as the first tone to each ear was presented simultaneously due to the rhythm being structured as a 3- versus 4-beat meter (see Fig. 1a). The configuration of which ear (left or right) received the specific tone streams (the slower or faster tempo) was counterbalanced across experimental blocks.

In each block, participants were instructed to attend to tones in either the left or the right ear and to respond with a button press (i.e. the spacebar on a computer keyboard) to SOA deviants (i.e. targets) in the attended ear. Participants used their dominant hand and were instructed to respond as quickly and accurately as possible. As the tempo of tones *within* the attended ear varied (slow vs. fast), the design permitted manipulation of spatial- as well as temporal attention. Each block consisted of regular and irregular sequences of tones (Fig. 1b). The irregular sequences contained one SOA deviant (−90 ms) in any of the 5 tones following the first tone, and the order of the irregular sequences (i.e. in which position the SOA deviant occurred) was randomized in each block. Having SOA manipulations for all tones in the sequence ensured that participants could not strategically prepare for one single tone in the sequence. Each block consisted of 50 regular sequences, and rhythm predictability was manipulated by varying the number of additional irregular sequences (containing a SOA deviant) between blocks. *High predictability* blocks had 10 (~17%) irregular sequences (2 SOA deviants for each tone except the first), while *low predictability* blocks had 25 (~33%) irregular sequences (5 SOA deviants for each tone except the first) (Fig. 1b). The order of regular and irregular sequences in each experimental block was semi-randomized, as a minimum of one regular sequence was presented between irregular sequences. The balance of SOA deviants presented to the attended (first number) and the unattended (second number) ear in each block depended on both predictability and attended tempo: high predictability, attend slow = 4/6, high predictability, attend fast = 6/4, low predictability, attend slow = 10/15, and low predictability, attend fast = 15/10. This resulted in a total of 70 SOA deviants being presented to the attended and unattended ears (20 and 50 per high and low predictability blocks, respectively), as 8 blocks were presented in total to achieve counterbalancing of attended ear (left vs. right), attended tempo (slow vs. fast), and predictability (high vs. low) (Fig. 1c).

High-predictability blocks lasted for 168 s each, and low-predictability blocks lasted for 204 s each (total task duration of 25 min excluding breaks). Task instructions were presented on a computer screen positioned 80 cm in front of the participants. To reduce eye-movements throughout the experimental blocks, participants fixated on a black cross presented in the middle of the screen against a gray background. Participants received feedback in terms of the percentage of the deviants in the to-be-attended ear (correct) and in the to-be-ignored (incorrect) ear they responded to, as well as how many responses (raw number) were given when no deviant was presented (incorrect). The feedback was presented on the computer screen after completion of each experimental block.

### Goldsmiths musical sophistication index

As the current study involved rhythmic stimuli, the Goldsmiths Musical Sophistication Index (Gold-MSI) questionnaire (Müllensiefen et al. 2014) was used to assess individual differences in the participants’ level of musical experience. This allowed us to investigate the potential association between musical experience and attention modulation effects. The Gold-MSI is employed as a nuanced measure of musical experience in a nonmusician sample. This self-report inventory includes a measure of *general musical sophistication*, which is computed based on a selection of items from the five subscales; (1) “active musical engagement”, (2) “perceptual abilities”, (3) “musical training”, (4) “singing abilities”, and (5) “sophisticated emotional engagement with music”. This measure was used to have a single measure per participant capturing the amount of musical training as well as other factors that contribute to a participant’s level of musical experience. Possible scores range from 18 to 126, and higher scores indicate an increased level of musical experience. The Gold-MSI questionnaire takes approximately 10 min to complete.

### Seashore rhythm test

Rhythm perception abilities (detect rhythm violation) as well as auditory tracking abilities (sustained selective attention) were crucial in the current study. The seashore rhythm test (Seashore et al. 1960; Reitan and Wolfson 1985) was used to assess the participants’ basic perception of auditory rhythm. It requires participants to discriminate between 30 like and unlike pairs of simple musical rhythms with three possible lengths (5, 6, or 7 notes). The order of rhythmic patterns is presented from the shortest to the longest (10 pairs of each fixed length). This assessment was done to ensure that participants included in the analysis fell within the normal range in terms of rhythm perception. Furthermore, the test is considered useful for examining concentration and tracking abilities, meaning that poor performance potentially reflects deficient rhythm perception and/or tracking abilities (Lezak 1995). The range of possible raw scores is from 0 to 30. The total duration of the Seashore Rhythm Test is approximately 10 min.

### Procedure

At the beginning of the session, participants performed a 10-min practice run of the Dichotic Listening task to get familiar with the stimuli and instructions. During practice, they listened to the tempo—ear configurations (slow_left_–fast_right_ and fast_left_–slow_right_, configurations are illustrated in Fig. 1c). Each configuration was played twice, once with instruction to tap along to the right-ear tones, and then to the left-ear tones. There was no tapping during the actual experiment. Additionally, a short task involving detecting SOA deviants in the attended ear and ignoring SOA deviants in the unattended ear was performed to make sure participants understood the nature of the task. After the practice run, participants performed 8 experimental blocks. Each block had 8 sequences at the beginning without SOA deviants: 4 monaural (unattended ear silenced) and 4 binaural. The purpose was to further practice on directing attention to the instructed ear and relevant tempo before introducing the experimental stimuli. There was a break between each block, and the participants initiated the next block when they were ready to continue. The order of the 8 experimental blocks was randomized for each participant (the distribution of block order is reported in Foldal et al. 2020). An entire session (with breaks), including all tasks and questionnaires, as well as experimental set-up, lasted for approximately 2.0–2.5 h.

### Analysis of behavioral data

Previous studies reported associations between behavioral performance and EEG dynamics in attentional selection, specifically, enhanced attentional modulation for trials with correct compared to incorrect behavioral responses (Haegens et al. 2011; Boncompte et al. 2016; Wöstmann et al. 2016). For the current study, the trials (rhythm sequences) of interest did not involve a behavioral response. As the experimental design did not permit identifying trials in which participants might have been disengaged from the task, the only way of controlling for potential task-disengagement was to assess participants overall performance in the Dichotic Listening task.

This assessment involved analyzing the participants’ response patterns. For each participant, we categorized the total number of responses (i.e. button presses) that was given throughout the experiment into three different response types; hits (responses to attended deviants, i.e. targets), spatial errors (SEs, responses to deviants in the unattended ear), and random errors (REs, responses when no deviant was presented in either the attended or the unattended ear). If a response was given within 2 s following a deviant presented to the attended or unattended ear, it was considered a response to the deviant (i.e. hits or SEs). Otherwise, responses were considered as REs. Note that both types of errors (SE and RE) may be considered false alarm responses. Nonetheless, as they are qualitatively different (i.e. responding to a SOA deviant vs. responding in absence of a SOA deviant), we decided to treat them as two different types of errors in the analysis. We then computed the proportion of each response type (hits, SEs, and REs) relative to the total number of button presses for each participant (for a similar approach, see Wöstmann et al. 2016). Thereby, we could assess whether the participants’ proportion of hits was greater than the two types of errors (i.e. SEs and REs), which would be expected if participants performed according to task instructions. As the response pattern serves as a behavioral read-out of attention allocation, it was therefore used for assessing its association with indices of attention modulation at the neural level, described further under “Statistical analysis” (“*Association between attention modulation strength and behavioral measures*”).

Performance according to task instruction was confirmed in our sample of participants (see Results). Accordingly, we proceeded with further analyses assessing to what extent performance was affected by rhythm predictability. Specifically, we investigated whether the ability to detect targets (i.e. deviants presented in the attended ear) was influenced by rhythm predictability. For each participant, the ability to detect targets was computed as the proportion of presented targets the participant responded to, which will be referred to as “target detection rate” (TDR). Importantly, this was computed for the high- and low-predictability conditions separately.

### E‌EG – recording and preprocessing

Continuous EEG and electro-oculography (EOG) data were recorded using a BioSemi Active Two 64 Ag-AgCl electrode system (BioSemi, Amsterdam, Netherlands). Electrodes were placed according to the International 10–20 system (Chatrian et al. 1985). Data were sampled at 1024 Hz during online recording. EOG electrodes were placed above and below the right eye, and lateral to the participant’s left and right eye. Additional electrodes were positioned on both earlobes for later re-referencing.

For offline EEG processing, we used the Fieldtrip toolbox (Oostenveld et al. 2011) for Matlab (R2018a, Mathworks Inc., Natick, MA, USA). A 0.5 Hz high-pass filter was applied to the continuous EEG data to remove slow drifts. Further, data were low-pass filtered at 90 Hz, and a band-stop filter at 50 Hz (±2 Hz) was used to remove line noise. The data were re-referencing to averaged earlobes for preprocessing and down-sampled to 512 Hz. Noisy segments of the continuous data and bad channels were identified by visual inspection, and removed before running an independent component analysis (ICA). A mean number of 3.3 bad channels (SD = 1.7) was identified across participants. The ICA was used to identify and then manually remove blinks and horizontal eye movements in the non-epoched data.

The EEG data were segmented into epochs from −1 to 3.5 s relative to onset (1st tone) of regular tone sequences, meaning that sequences containing SOA deviants were excluded from the EEG analysis. Regular sequences for which the previous sequence contained a SOA deviant (i.e. an irregular sequence) were also excluded from the analysis. This was done to avoid potential confounding effects resulting from the processing of- and motor response to the SOA deviant in the preceding sequence. Additionally, regular sequences during which participants gave a response even when a deviant had not occurred (i.e. a random error) were rejected for the same reason. This procedure resulted in ~40 sequences per high-predictability block, and ~25 sequences per low-predictability block. Note that these numbers differ from the number of 50 regular sequences presented in both high- and low-predictability blocks (see Fig. 1b). Epochs were rejected based on previously defined noisy segments in the continuous EEG data if the defined noisy segments overlapped with a time-window of 3.5 s around sequence onset (−0.5 to 3.0 s). The resulting mean number of epochs across participants for the different experimental conditions is presented in Table 1. Excluded bad channels were reconstructed using spherical spline interpolation (Perrin et al. 1989), and the data were rereferenced to a common average of all 64 EEG-channels. Epochs were detrended and demeaned by subtracting the average amplitude over a time-window spanning −0.2 to 2.2 s relative to rhythm sequence onset.

**Table 1:** The mean number of trials across participants per experimental condition after EEG preprocessing.

High predictability				Low predictability			
Attend left		Attend right		Attend left		Attend right	
Slow	Fast	Slow	Fast	Slow	Fast	Slow	Fast
36.7 (2.0)	36.7 (2.3)	37.2 (2.7)	36.7 (2.0)	23.6 (1.6)	23.4 (1.5)	22.5 (2.9)	23.1 (1.8)

Standard deviations are given in parentheses.

### Analysis of attention modulation and effects of rhythm predictability

The time–frequency representation of power for the single trials (i.e. tone sequences) was obtained via wavelet transform, using Morlet wavelets with a width of seven cycles from 1 to 90 Hz (in steps of 0.5 Hz and 50 ms).

#### E‌EG channel and frequency band selection

Frequency band and channel selection was performed on a time-window separate from that used for statistical analyses (Haegens et al. 2011; van Ede et al. 2011). Specifically, −200 to 0 ms preceding each rhythm sequence was used for this purpose (“presequence”), while time 0–2.2 s of the rhythm sequences was used for statistical analyses. The time between the onset of each sequence was 2.4 s, meaning no overlap between these time-windows. Each participant’s trials were averaged for the “attend left” and “attend right” conditions (across “attended tempo” and “rhythm predictability”). The normalized attention modulation index (AMI) was calculated as follows:

(1) \begin{equation*} \mathrm{AMI}=\frac{\ \left(\mathrm{attend}\ \mathrm{left}\right)-\left(\mathrm{attend}\ \mathrm{right}\right)}{\ \left(\mathrm{attend}\ \mathrm{left}\right)+\left(\mathrm{attend}\ \mathrm{right}\right)} \end{equation*}

The procedure reduces variability in the power estimates between participants, and is therefore considered convenient for normalization purposes (Haegens et al. 2011; Wilsch et al. 2020). Resulting negative values indicate stronger power decrease during “attend left”-conditions, and resulting positive values indicate stronger power decrease during “attend right”-conditions. Similar to other sensory modalities (i.e. visual and somatosensory perception), the auditory system has stronger contralateral than ipsilateral sensory pathways (Rosenzweig 1951; Tervaniemi and Hugdahl 2003). Accordingly, contrasting “attend left” and “attend right” conditions is assumed to result in a topography of power values that is generally negative over the right hemisphere channels (beta suppression during “attend left”), and positive over the left hemisphere channels (beta suppression during “attend right”).

The presequence interval was used to establish the presence of attention modulation effects, and as a means to extract relevant features (e.g. electrodes and frequencies) for the following analyses focusing on the time-window of interest (0–2.2 s), by contrasting the normalized “attend left” and “attend right” conditions (e.g. Haegens et al. 2011; Haegens et al. 2012; Wilsch et al. 2020). Cluster-based permutation tests (Maris and Oostenveld 2007) were used, randomly swapping the labels of the conditions (10,000 iterations). This was performed for the alpha (7–13 Hz)- and the beta (14–24 Hz)-band separately, which permitted identification of the frequency band of interest (beta-band). Since no significant attention modulation (AMI) was found for the alpha-band during the presequence time-window, this frequency band was not considered for further analysis steps (Supplementary Fig. S1A). The results of the cluster-based permutation tests were also used to decide on the number of channels to be selected for further analyses. Previous studies on attentional modulation of alpha-beta power lateralization typically selected a subset of left and right hemisphere channels for statistical analyses (e.g. Haegens et al. 2011; Wöstmann et al. 2016; Heideman et al. 2018a; Wilsch et al. 2020). For the present study, we decided on the number channels to include based on averaging the number of channels in the first positive (left hemisphere) and the first negative (right hemisphere) cluster identified for the beta band in the presequence time-window (i.e. 10 channels). Based on the grand average AMI topography (across “tempo” and “predictability” conditions, −200 to 0 s), channels were sorted by AMI-values. A left ROI was created by selecting the 10 channels with the most positive AMI values over the left hemisphere, while the 10 channels with the most negative AMI values over the right hemisphere were used to create a right ROI, excluding midline channels (Supplementary Fig. S1A).

#### Scalp-level analyses

Following identification of channels (ROIs) as well as the frequency band (beta) to be used for further analyses, attentional modulation of neural responses was calculated for each time–frequency bin spanning the length of the rhythm sequences (0–2.2 s) for each channel. Attention modulation was calculated as described above (AMI), based on each participant’s mean 14–24 Hz power, across “attend left” and “attend right” trials, for each rhythm predictability condition (“low” vs. “high”) separately. Finally, the average left-hemisphere (left ROI) and right-hemisphere (right ROI) AMI values, for both predictability conditions, were computed for each participant. As the analysis of attention modulation was performed on the average power across the rhythm sequences (0–2.2 s), thereby not addressing the temporal dynamics, this analysis involved combining data from the two “attended tempo”-conditions (“attend fast” and “attend slow”).

#### Source-level analysis

Effects of attention modulation were also analyzed at source-level. Importantly, the same procedure as for the scalp-level data to define a left and right ROI in source-space was applied. We performed dynamic imaging of coherent sources (DICS; Gross et al. 2001) beamforming for the beta band (19 Hz with ±5 Hz spectral smoothing) averaged across the presequence interval (−0.2 to 0 s), in order to select ROI grid points. For each participant, a common spatial filter was constructed from the cross-spectral density matrix of the scalp EEG signal from all trials combined (“attend left” and “attend right” trials). Scalp-level power from the “attend left” and the “attend right” conditions was then mapped onto source-space using the common filter. We used a three-layer boundary element model (BEM) of an MNI template brain MRI (Colin27) provided by the Fieldtrip toolbox (Oostenveld et al. 2011) with a 5 mm spaced source grid. A regularization parameter (lambda) of 5% was applied.

The source-estimated power for the “attend left” and “attend right” conditions was normalized in the same way as scalp-level data (see above section: “EEG channel and frequency band selection”), and contrasted using a cluster-based permutation test, to confirm the frequency band of interest (beta-band) and establish the number of grid-points to be used for a left- and right-ROI in source space. Again, this number was computed by averaging the size (i.e. number of grid-points) of the first identified positive (left lateralized) and first negative (right lateralized) cluster (i.e. 118 grid points, per left and right ROI). As with the scalp-level data, the grand average AMI (normalized difference between “attend left” and “attend right” conditions) was computed across subjects for the presequence interval. Grid points were sorted by AMI-value, and a left ROI was defined using the grid-points with the most positive AMI-values from the left hemisphere as well as a right ROI based on grid-points with the most negative AMI-values from the right hemisphere (midline grid-points excluded). For visualization of clusters and resulting ROIs at source level, see Supplementary Fig. S1B.

Using the same parameters as above, we performed the DICS on beta-band power across the rhythm sequence (0–2.2 s), but now splitting the data by rhythm predictability (“low” vs. “high”). Again, scalp-level power from the “attend left” and the “attend right” condition, for high and low rhythm-predictability separately, was mapped onto source-space using a common spatial filter. Then, the average left- and right ROIs AMI values, for both predictability conditions, were computed for each participant.

### Analysis of time-resolved lateralization

The AMI reflects the topographical representation of attention modulation, and is based on a contrast between different conditions (attend left vs. attend right). For this reason, it does not provide information about how this lateralization evolves over time during both “attend left” and “attend right” trials, which was also a central aim of the present study. To obtain a time-resolved measure of hemispheric lateralization, attend-left and attend-right trials were first separated according to the attended tempo (slow vs. fast) resulting in four conditions. Note that at this point, data from the two rhythm predictability conditions (high and low) were combined. The first reason was to obtain a sufficient number of trials per condition, as splitting data according to both attended tempo and rhythm predictability would result in a low trial-count (i.e. less than 30 trials for low-predictability conditions). Second, it is argued that phase-based analyses are more sensitive to differences in the number of trials between conditions compared to power-based analyses (Cohen 2014), which was the case for the present data where the trial-count was greater for the high- compared to low-predictability conditions.

At scalp-level, power was already time-resolved based on the time–frequency analysis that had been conducted. To obtain a time-resolved source signal, the DICS was recomputed for the beta band (19 Hz) in steps of 50 ms over the time-course of the sequence (−0.15 to 2.35 s). The length of the moving window was set to 300 ms (±150 ms) to obtain the desired spectral smoothing of ±5 Hz, as well as accommodating at least 3 cycles for the lower frequencies in the spectral band of interest (14–24 Hz). For each time-bin, we computed a common spatial filter based on the cross-spectral density matrix of the scalp EEG signal from all trials combined (“attend left – slow”-, “attend right – slow”-, “attend left – fast”-, and “attend right – fast”-trials). The time-resolved scalp-level power from each condition was then mapped onto source space using the common spatial filter.

While the analysis described above concerning the topographical representation of attention modulation (i.e. AMI), was based on common left and right ROIs across subjects, individually selected ROIs (based on each individual’s grand average AMI across experimental conditions) were used for the analysis of the time-resolved lateralization. The size of the ROIs (i.e. number of channels and grid-points) was the same as for the AMI-analysis described above. The procedure of using individual ROIs for analyzing the temporal progression of lateralization effects has also been used in previous studies, as the spatial topography of attention modulation effects can vary between individuals (Haegens et al. 2011; Wöstmann et al. 2016). This was also the case in the present study, and a visualization of the selected channels and sources across participants is presented in Supplementary Fig. S2 (Supplementary material). For each participant and each condition, the time-resolved beta-power from the left and right hemisphere ROIs were defined either as ipsi- or as contra-lateral depending on the direction of attention for that specific condition (i.e. for attend left ear conditions, the left ROI beta was considered ipsilateral, while the right ROI beta was considered contralateral, etc.). Prior to computing the time-resolved lateralization index (LI), at both scalp-level and source-level, a centered moving average window (250 ms) was applied to each signal (ipsilateral and contralateral) for smoothing purposes. The average ipsilateral and contralateral time courses were then computed across the “attend left” and “attend right” conditions, for “attend slow” and “attend fast” separately. In this way, the LI could be computed and normalized as follows for each time-bin;

(2) \begin{equation*} \mathrm{LI}=\frac{\ \left(\ \mathrm{power}\ \mathrm{ipsi}\right)-\left(\mathrm{power}\ \mathrm{contra}\right)}{\ \left(\mathrm{power}\ \mathrm{ipsi}\right)+\left(\mathrm{power}\ \mathrm{contra}\right)} \end{equation*}

It was computed for the “attend slow” and “attend fast” conditions separately. An increase in the LI (more positive) suggests stronger lateralization, namely suppressed beta-power in areas contralateral to the attended side relative to areas ipsilateral to the attended side.

### Spectral analysis of the time-resolved lateralization index

Fast Fourier Transforms (FFTs) were calculated at 1.25 and 1.66 Hz for each participant’s LI time courses (“attend slow” and “attend fast”), for both scalp-data and source-data. Zero-padding was applied to obtain a frequency resolution of 0.01 Hz. First, FFTs were computed at 1.25 Hz resulting in two conditions; “1.25 Hz attended” (for the “attend slow” LI time-courses) and “1.25 Hz unattended” (for the “attend fast” LI time-courses). Second, FFTs were computed at 1.66 Hz resulting in two conditions; “1.66 Hz attended” (for the “attend fast” LI time-courses) and “1.66 Hz unattended” (for the “attend slow” LI time-courses). In other words, FFTs at 1.25 and 1.66 Hz could be analyzed in terms of representing the attended or unattended tempo. For each participant, the phase angles for each frequency when representing the attended as well as the unattended tempo were computed; phase angle 1.25 Hz – attended, phase angle 1.25 Hz – unattended, phase angle 1.66 Hz – attended, and phase angle 1.66 Hz – unattended.

### Statistical analysis

The normality of all data variables (behavioral and electrophysiological) was evaluated using the Shapiro-Wilks test (Shapiro and Wilk 1965) and visual inspection of Normal Q–Q plots. Parametric tests were used when variables met the assumption of approximate normal distribution. Otherwise, nonparametric tests were used. However, the investigation of interaction between attention modulation (left vs. right hemisphere AMI) and rhythm predictability (high vs. low) required the use of two-way repeated measures ANOVAs. Approximate normal distribution of variables included in the ANOVAs was ensured by computing the *z*-scores for each variable to identify and exclude participants (*n* = 2) exhibiting *z*-scores greater than 3.0 (considered outliers). Accordingly, the sample used for analysis included data from 30 participants.

#### Behavioral data

A Friedman test was used to assess the proportion of the different response types (i.e. hits, SEs, and REs). Follow-up Wilcoxon signed-ranks tests were used to assess the difference between the proportion of hits and the two error types. A paired *t*-test was employed to investigate the difference in “TDR” between the low- and high-predictability conditions.

#### AMI

To investigate attention modulation and effects of rhythm predictability at the neural level, we conducted repeated measures ANOVAs (scalp- and source-level data), with hemisphere (left vs. right) and rhythm predictability (low vs. high) as within-subject factors. We also report the effect size omega squared (ω^2^), which is interpreted as follows; >0.14 = large effect; 0.06–0.14 = medium effect; 0.01–0.06 = small effect (Kirk 1996). The effects of attention modulation and rhythm predictability were additionally assessed within a Bayesian framework. The Bayes Factor (BF) is an index of the relative support for one hypothesis (or model) relative to another (Wagenmakers et al. 2018b), and we report the BF as a measure of strength of evidence in favor of the alternative hypothesis (H1) relative to the null hypothesis (H0). It is computed as the ratio of the probabilities for each hypothesis given the data:

(3) \begin{equation*} \mathrm{BF}=\frac{\mathrm{probability}\ \left(\mathrm{H}1\ |\ \mathrm{data}\right)}{\mathrm{probability}\ \left(\mathrm{H}0\ |\ \mathrm{data}\right)} \end{equation*}

Hence, BF *>* 1 favors the H1, while BF < 1 favors the H0. Recommendations for assessing the strength of evidence are as follows; 0.33–1 or 1–3 (anecdotal evidence), 0.10–0.33 or 3–10 (moderate evidence), and < 0.1 or > 10 (strong evidence) (Dienes 2014; Lee and Wagenmakers 2014). The BF represents a model comparison, and we were interested in whether an interaction-effect model represents the data better compared to a main-effect model, or the other way around. Hence, the resulting BFs for interaction effects presented in this article reflect a comparison between a model that includes the interaction term and one that includes terms for main effects only (for a more comprehensive explanation of computing Bayes Factors for the comparison of two-way ANOVA models, see Wagenmakers et al. 2018a; Vijayaragunathan and Srinivasan 2020).

#### Time-resolved LI

Statistical analysis of the time-resolved LI was analyzed in two steps to assess potential differences in phase angle between experimental conditions, and to assess the magnitude of phase consistency (i.e. phase concentration) at the two frequencies of interest within experimental conditions: 1) A two-factor ANOVA for circular data was conducted (Harrison-Kanji test; Harrison and Kanji 1988), to determine whether there was a difference in mean phase angle depending on whether a frequency represented the attended or the unattended tempo, including the two factors; *frequency* (1.25 vs. 1.66 Hz) and *attention* (attended vs. unattended). The Harrison-Kanji test can be viewed as a circular version of the two-factor ANOVA, where the reported statistic depends on the kappa of the von Mises distribution (a circular normal distribution) applied to the data. The Chi-squared statistic (χ^2^) is reported when the kappa value is low (<2) while the F-statistic is reported for higher kappa values. If the phase angle of a specific frequency differs depending on whether it represents the attended or unattended tempo (e.g. phase opposition) during both “attend slow” and “attend fast” conditions, a main effect of attention would be expected. However, the alignment of beta-lateralization at a particular phase angle should not change depending on whether stimuli are presented with a slightly slower or faster tempo. Hence, a main effect of frequency or interaction with attention was not expected. 2) Rayleigh tests (see Fisher 1995) were used to investigate the consistency of 1.25 and 1.66 Hz phase angles across subjects in terms of whether they represented the “attended” or “unattended” tempo. In other words, we would expect 1.25 Hz phase angles to be more consistent compared to 1.66 Hz phase angles across subjects during the “attend slow” condition. Vice versa, we would expect 1.66 Hz phase angles to be more consistent compared to 1.25 Hz phase angles across subjects during the “attend fast” condition. More consistent phase angles for the frequency representing the attended tempo suggest that fluctuations in the lateralization effects synchronize more strongly with that specific tempo. Differences in the consistency between 1.25 and 1.66 Hz phase angles within the “attend slow” and the “attend fast” condition were further assessed by means of permutation tests. The discrepancy in phase consistency was computed as the difference between the mean resultant vector length of 1.25 Hz phase angles and the mean resultant vector length of 1.66 Hz phase angles, for “attend slow” and “attend fast” conditions separately. This difference measure was compared against a null-distribution computed by randomly permuting the condition labels (“1.25 Hz” and “1.66 Hz,” 10,000 iterations). Statistical analyses were performed for both scalp- and source-data.

#### Association between attention modulation strength and behavioral measures

Finally, we were interested in whether there was an association between attention modulation at the neural level and performance in the Dichotic Listening task. For each participant, attention modulation strength was computed as the mean difference in AMI values between the left and right hemispheres. Pearsons’s or Spearman’s rank correlation coefficients were computed depending on whether assumptions of normality were met. As we had specific hypotheses regarding the direction of effects, one-tailed tests were used. We expected increased attention modulation strength to be associated with an increased proportion of hits, and a reduced proportion of SE- and RE-responses (i.e. improved performance).

## Results

### Behavioral measures

Scores on the Seashore Rhythm test (mean, 28.2; range, 25–30, SD, 1.4) revealed no apparent deficiency in rhythm perception or tracking ability for any of the participants. Furthermore, their Gold-MSI scores (mean, 66.0; range, 35–107, SD, 17.6) were in accordance with what would be expected in a nonmusician sample of young healthy adults (Müllensiefen et al. 2014; Baker et al. 2020).

**Fig. 2:**
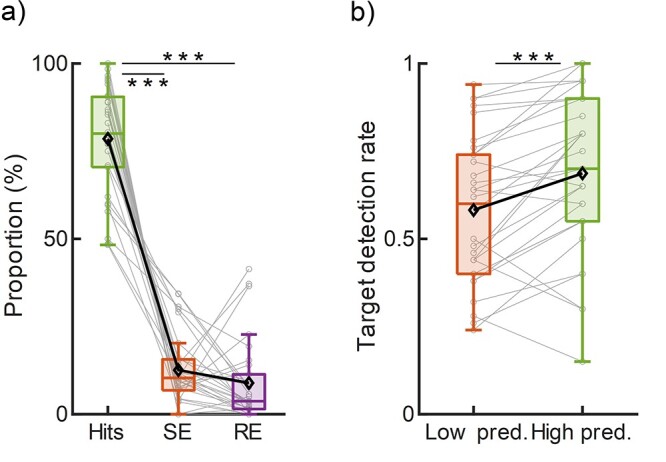
Behavioral performance on the dichotic listening task. (a) Boxplot depicting the proportion (%) of hits, spatial error (SE), and random error (RE) responses relative to the total number of responses throughout the entire experimental session. (b) Boxplot depicting the rate of detected deviants relative to the number of deviants presented to the attended ear (“target detection rate”) in the low- and high-predictability (pred.) conditions. In each boxplot (a, b), the upper and lower box edges represent the 25th and 75th percentile, respectively, and whisker length is equal to 1.5 times the interquartile range. The middle line in each box represents the median, and the mean for each condition in each plot is highlighted with a black diamond. Gray lines are drawn between pairs of individual data points. ^*^^*^^*^P < 0.001.

Analysis of the overall response pattern in the Dichotic Listening task (Friedman test) revealed that the proportions of each response type (i.e. hits, SEs, and REs) were significantly different, χ^2^(2, *n* = 30) = 47.41, *P <* 0.001. Wilcoxon signed-ranks tests showed that the proportion of hits was larger compared to both the proportion of SE responses, *Z* = −4.82, *P <* 0.001, and RE responses, *Z* = −4.82, *P <* 0.001. This suggests that the participants were able to perform according to the task instruction (Fig. 2a). We further investigated how rhythm predictability influenced participants’ ability to detect deviants in the attended ear (“TDR”). As a SOA deviant (−90 ms) was proportionally a more salient deviant in the “attend fast” compared to the “attend slow” condition, we checked that there was no significant difference in participants ability to detect deviants in the attended stream between these two conditions. The results revealed a significantly higher “TDR” in the high- compared to the low-predictability condition, *t*(29) = 4.28, *P <* 0.001 (Fig. 2b). Note that the low-predictability condition involved presentation of more targets. Accordingly, a missed target in the high-predictability condition (e.g. due to lapses of attention) has a greater impact on the detection rate compared to the low-predictability condition. One should therefore exert caution when interpreting the behavioral effect of rhythm predictability. Importantly, the number of tone sequences that did *not* contain deviant intervals was the same for low- and high-predictability blocks, which were used for analysis of the electrophysiological data.

### Electrophysiological measures

#### Attention modulation and effects of rhythm predictability

For the analysis of attention modulation, the frequency band of interest (beta, 14–24 Hz) and a left and a right hemisphere ROI (at scalp- and source-level) had been defined based on a cluster-based permutation analysis performed on an independent presequence time-window (see Method section and Supplementary Fig. S1 for details).

**Fig. 3:**
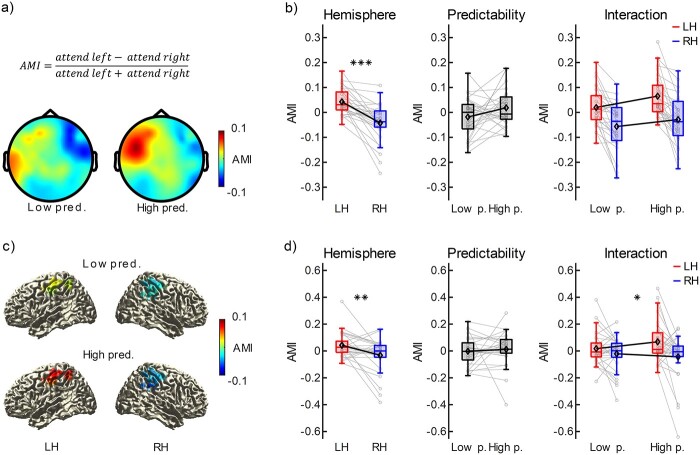
Grand average AMI topographies (scalp and source level) for the two predictability conditions and results from the rmANOVAs. (a, c) Topographies, grand average AMI at scalp-level (a) and source-level (c), low vs. high predictability. For source-level data (c) the masks illustrate the left and right hemisphere ROIs. (b, d) Boxplots depicting the AMI for each condition at scalp-level (b) and source-level (d), illustrating the main effects of hemisphere (left), main effects of predictability (middle), and the interaction between hemisphere and predictability (right). In each boxplot, the upper and lower box edges represent the 25th and 75th percentile, respectively, and whisker length is equal to 1.5 times the interquartile range. The middle line in each box represents the median, and the mean for each condition in each plot (left, middle, and right) is highlighted with a black diamond. Gray lines are drawn between pairs of individual data points. ^*^^*^^*^P < 0.001, ^*^^*^P < 0.01, ^*^P < 0.05.

The defined ROIs were used to analyze attention modulation of beta-power and effects of rhythm predictability across the rhythm sequence (0–2.2 s) at scalp-level. The grand average AMI values over the scalp are presented in Fig. 3a. The repeated measures ANOVA revealed a significant main effect of hemisphere (left vs. right ROI), *F*(1,29) = 31.61, *P <* 0.001, *ω*^2^ = 0.293 (BF = 1.08e+5, strong evidence for a difference), reflected as positive AMI-values over the left ROI and negative AMI-values over the right ROI. However, neither the main effect of rhythm predictability (high vs. low predictability), *F*(1,29) = 3.15, *P =* 0.086, *ω*^2^ = 0.038 (BF = 1.61, anecdotal evidence for a difference), nor the interaction between hemisphere and predictability, *F*(1,29) = 1.97, *P =* 0.171, *ω*^2^ = 0.003 (BF = 0.81, anecdotal evidence against an interaction), were significant. Box plots representing each effect from the ANOVA (hemisphere, predictability, and interaction), including group means and individual data points, are presented in Fig. 3b. In sum, the results indicate a clear presence of attention modulation, reflected as positive AMI values over the left-, and negative AMI values over the right hemisphere. The complementary BF-analysis provides strong evidence for attention modulation, while the effect of predictability and its interaction with attention modulation remains inconclusive (i.e. anecdotal evidence only).

Visual inspection of the time–frequency representation of the AMI for left and right hemisphere channels (Supplementary Fig. S3) suggests that attention modulation effects might not be restricted to the beta-band, as it extends into gamma-band frequencies (>30 Hz). Muscle activity can be represented in frequencies as low as 20 Hz (for a review, see Muthukumaraswamy 2013), and based on the topography of gamma-band (55–75 Hz) power (see Supplementary Fig. S4A, Supplementary material), we suspected that it potentially reflected cranial muscle- and not cortical activity (Hipp and Siegel 2013). Source analysis of EEG data, using Beamformer methods, has been shown to efficiently account for activity resulting from noncortical sources (Hipp and Siegel 2013). Therefore, we applied a DICS Beamformer and additionally performed the analysis of beta-power at source-level to ensure that effects represent modulations of cortical activity, and not influence from muscle activity “leaking” into the beta band. Using the same procedure as with the scalp-data, a left and a right hemisphere ROI at source-level had been defined based on data from the presequence time-window. The ROIs were used to analyze attention modulation of beta-power and effects of rhythm predictability across the rhythm sequence (0–2.2 s) at source-level. The grand average AMI values over the left and right ROIs in source-space are presented in Fig. 3c. Similar to the scalp-level analysis, the main effect of hemisphere at source-level was significant, *F*(1,29) = 8.06, *P =* 0.008, *ω*^2^ = 0.091 (BF = 13.45, strong evidence for a difference), and the main effect of predictability was not, *F*(1,29) = 0.24, *P =* 0.626, *ω*^2^ = 0.000 (BF = 0.22, moderate evidence against a difference). On the other hand, the interaction between hemisphere and predictability was statistically significant at source-level, *F*(1,29) = 4.31, *P =* 0.047, *ω*^2^ = 0.027 (BF = 0.74, anecdotal evidence against an interaction). Box plots representing each effect from the ANOVA at source-level (hemisphere, predictability, and interaction), including group means and individual data points, are presented in Fig. 3d. The effects remained similar at source-level, indicating a clear presence of attention modulation (i.e. hemisphere difference). This was supported by the complementary BF-analysis, suggesting strong evidence for a difference between hemispheres, despite the effect size being reduced in comparison to the scalp-level data. Furthermore, the results suggested moderate evidence against an overall effect of predictability. The interaction between attention modulation (hemisphere difference) and predictability (high vs. low) was reflected as enhanced attention modulation during the high- compared to the low-predictability condition. However, the effect was small in terms of effect size, and the BF indicated inconclusive results (i.e. anecdotal evidence only). Finally, comparing the left- and right-hemisphere AMI-values for the gamma-band (55–75 Hz) at scalp- and source-level, suggested that the effect was reduced at source-level (see Supplementary Fig. S4B). Also, there was no clear pattern of both positive values over the left and negative values over the right hemisphere for the gamma-band at source-level, as AMI values over the right hemisphere tended to center around zero (i.e. no difference between “attend left” and “attend right” conditions). It is therefore unlikely that the effects demonstrated for the beta-band were solely driven by gamma-lateralization effects leaking into the beta-band.

Notably, while assessment of the overall behavioral performance across participants suggested performance according to task instruction, the variability was relatively high. In particular, the proportion of RE-responses (responding when no deviants presented) ranged between 0 and 40%. We suspected that participants with a high proportion of such errors were not paying attention as instructed, or had great difficulty with the task, which might further introduce noise in the data. To address this, we assessed whether the degree of attention modulation (left vs. right hemisphere difference) in each participant predicted their proportion of RE responses. A robust linear regression was computed and revealed that degree of attention modulation predicted the proportion of random errors, *R*^2^ = 0.27, *F*(1,28) = 10.52, *P =* 0.003. The effect reflected that the proportion of random errors decreased as attention modulation increased (see Supplementary Fig. S5 for a visualization of this result). Participants with a high number of random errors would also have a higher number of excluded “regular sequence”-trials (which were used for the EEG-analyses), and an imbalance in trial-count is known to have a greater impact on phase-based compared to power-based analyses (Cohen 2014). We therefore decided to exclude participants with the most extreme proportions of RE (>20%, *n* = 4) for the remaining analyses to reduce potential noise (i.e. from inattention or difficulty with the task) and to avoid issues related to differences in trial-count (i.e. for the remaining phase-based analysis of the time-resolved beta-power lateralization). In addition, we performed a reassessment of the reduced sample (*n* = 26) by computing the ANOVAs at scalp- and source-level, which revealed highly similar results (see “Attention modulation and effects of rhythm predictability” and Supplementary Fig. S6 in the Supplementary Material).

#### Temporal dynamics of attentional modulation

**Fig. 4:**
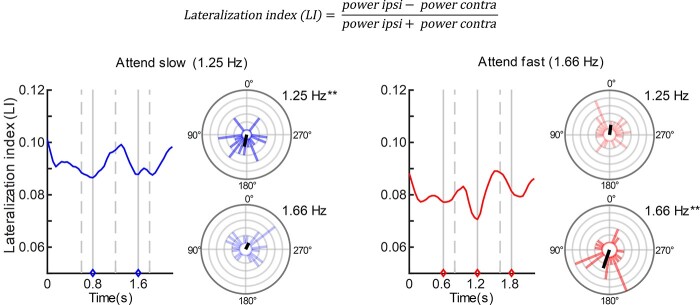
Time-courses of the LI and distribution of 1.25 and 1.66 Hz phase angles for scalp-level data during the “attend slow” (left – blue color) and “attend fast” (right – red color) conditions. For the time-courses, tone onsets are depicted as vertical gray lines marked as either attended (solid) or unattended (dashed) according to the attended tempo (slow vs. fast). Attended tones are additionally highlighted with diamonds on the time-axis. The distribution of both 1.25 and 1.66 Hz phase angles are depicted next to each time-course, where the phases of the frequency representing the attended tempo (1.25 or 1.66 Hz) have a darker shaded color. The black line represents the mean resultant vector. ^*^^*^P < 0.01 (Rayleigh test).

The LI represents attention modulation of beta power over time (0–2.2 s). Larger LI values (positive) suggest increased beta power over areas ipsilateral to the side of attention relative to contralateral areas. Participants attended to stimuli presented at 1.25 Hz (slow tempo, SOA: 800 ms) or 1.66 Hz (fast tempo, SOA: 600 ms) depending on the direction of attention and the dichotic configuration of tones. The time-courses of the LI during the “attend slow” and “attend fast” conditions at scalp level are presented in Fig. 4. To determine whether attention synchronized with the tempo of the attended stream, FFTs at 1.25 and 1.66 Hz were computed for the LI time-courses. According to the tempo of the attended stream (i.e. slow vs. fast), these frequencies could be defined as either *attended* or *unattended.* For scalp-level data, results of the circular ANOVA revealed a significant main effect of attention, χ^2^(2) = 22.91, *P <* 0.0001. The effect is illustrated in the phase plots of Fig. 4, showing how the phase angles of the frequency representing the attended tempo (darker color) clustered around 180°, while the phase angles of the frequency representing the unattended tempo (lighter color) clustered around 0°, during both the “attend slow” and “attend fast” condition. Accordingly, there was no significant effect of frequency, χ^2^(2) = 0.12, *P =* 0.942, and no interaction between attention and frequency, χ^2^(1) = 0.29, *P =* 0.592. The results suggest that the mean phase angles of the lateralized beta power modulation align differently to the tempo of the attended versus unattended stream. This effect applied to both tempos or frequencies that were investigated.

We then assessed the consistency of 1.25 and 1.66 Hz phases extracted from the time-resolved lateralized beta-power modulation (LI) across participants. Specifically, we assessed whether phase consistency of the two frequencies changed depending on whether participants attended the slower or the faster tempo. At scalp-level, Rayleigh tests revealed that the phase consistency was significant for both 1.25 Hz, *P =* 0.008, and 1.66 Hz, *P =* 0.006, when these represented the attended tempo (see phase plots with darker shaded color in Fig. 4). However, when representing the unattended tempo, phase consistency of neither 1.25 nor 1.66 Hz was significant, *P =* 0.140 and *P =* 0.385, respectively (see phase plots with lighter shaded color in Fig. 4). Furthermore, we addressed whether the phase consistency of the frequency representing the attended tempo was significantly stronger compared with that of the unattended tempo within the “attend slow” and “attend fast” conditions. Permutation tests revealed that the difference in consistency (i.e. the difference in mean resultant vector length between the frequency representing the attended and the unattended tempo) was significant during the “attend slow” condition, *P =* 0.018, and at trend-level for the “attend fast” condition, *P =* 0.064. While the resulting power-spectrum from the FFTs performed on the “attend slow” and “attend fast” LI-time courses did not indicate clear peaks at 1.25 and 1.66 Hz, respectively (see Supplementary Fig. S7A), there was a clear shift in phase-consistency from the lower (i.e. around 1.25 Hz) to higher (i.e. around 1.66 Hz) frequencies depending on the frequency representing the attended tempo (see Supplementary Fig. S7B). In sum, these results indicate that the LI, reflecting neural attention mechanisms, appears to synchronize more strongly with the attended than the unattended tempo. Note that we also performed the LI-analyses for the gamma-band data, since we found clear lateralization of gamma-band power at scalp-level. Importantly, there was no indication that lateralization of gamma-band power synchronized significantly with the tempo of the attended or unattended sounds.

The LI-analyses (i.e. the circular ANOVA and Rayleigh tests) were also computed at source-level data, where effects were overall weaker and did not turn out significant (see Supplementary Fig. S8 for a visualization of the results at source level). The circular ANOVA revealed no significant effects of attention, χ^2^(2) = 4.97, *P =* 0.083, frequency, χ^2^(2) = 0.33, *P =* 0.848, or interaction between attention and frequency, χ^2^(1) = 0.10, *P =* 0.757. Furthermore, Rayleigh tests showed no significant phase consistency across participants for 1.25 Hz, *P =* 0.429, nor for 1.66 Hz, *P =* 0.240, when these represented the attended tempo. Similarly, when representing the unattended tempo, phase consistency of neither 1.25 nor 1.66 Hz was significant, *P =* 0.512 and *P =* 0.940, respectively.

#### Links between EEG indices of attention modulation and behavioral measures

Finally, we assessed the potential association between attention modulation strength, reflected in beta band dynamics, and performance on the Dichotic Listening task. Since hemispheric lateralization was stronger at scalp- compared to source-level, attention modulation strength at scalp-level was used for correlation analyses. We computed the attention modulation strength as the difference between the left and the right hemisphere (i.e. ROIs) AMI values across all other conditions. Correlation coefficients and *P*-values are presented in Table 2. Attention modulation strength did not correlate significantly with any of the behavioral measures. However, there was a trend towards increased attention modulation strength being associated with an increased proportion of hits (positive correlation) combined with a reduced proportion of SEs (negative correlation), but not with the proportion of REs nor TDR. In the Foldal et al. (2020) paper, which was based on the same data, we reported that participants with an increased level of musical experience (Gold-MSI) also performed better in the Dichotic Listening task. However, in that study only deviance response rates were analyzed. Our current extended analysis showed that participants’ level of musical experience correlated significantly with their proportion of hits, REs and TDR, but not with their proportion of SEs. The ability to selectively allocate attention is indicated in the relative share of hits and SEs, as this reflects how well participants distinguished between deviants presented to the attended and unattended ear. On the other hand, participants’ general sensitivity to SOA deviants in the rhythmic stimuli should be reflected in their proportion of hits, REs and TDR, as these measures indicate how well they evaluated sound timing. Hence, it appeared that the neural measure of attention modulation was to a greater extent associated with a behavioral readout of attention modulation (proportion of hits and SEs), while musical experience seemed to be associated with the ability to evaluate sound timing (hits, REs and TDR). Also, we found no significant correlation between attention modulation strength at the neural level and degree of musical experience (Pearsons’ *r* = 0.02, *P =* 0.454), supporting that our neural measure of attention modulation (AMI) and the measure of musical experience are associated with different performance readouts.

**Table 2:** Correlations between behavioral measures and beta-band attention modulation strength during the dichotic listening task, and self-reported musical experience.

	Attention modulation strength		Gold-MSI	
	*r*	*P*-value	*r*	*P*-value
Hits	0.32	0.056	0.44	0.012
SEs	− 0.31	0.060	−0.19	0.174
REs	−0.17	0.198	−0.38	0.029
TDR	0.26	0.103	0.64	<0.001

SEs = spatial error, REs = random error, TDR = target detection rate, Gold-MSI = Goldsmiths Musical Sophistication Index. Spearman’s correlation coefficients (one-tailed) are reported for association with the proportion of each response type (i.e. hits, SEs and REs), while Pearson’s correlation coefficients (one-tailed) are reported for associations with TDR.

## Discussion

We investigated attentional modulation of alpha- and beta-power while participants listened to dichotically presented rhythmic streams of tones. The rhythmic stimuli were presented in blocks that varied in terms of rhythm predictability. Participants responded to SOA deviants which were inserted rarely (high-predictability blocks) or more frequently (low-predictability blocks). Behavioral analyses showed that participants performed the task according to instructions (i.e. larger proportion of hits compared to either error type), and that their ability to detect SOA deviants (i.e. TDR) was enhanced during high- compared to low-predictability conditions. Analyses of electrophysiological data revealed that directing attention to a specific ear resulted in enhanced beta-band power in EEG channels and sources ipsi- versus contra-lateral to the direction of attention (i.e. right vs. left ear), and the attention modulation was more prominent in high- compared to low-predictability conditions. Crucially, analyses of the time-resolved lateralization showed that attentional modulation of beta-power aligned with the timing of attended ear tones in the presence of a concurrent irrelevant sound stream with different timing. Finally, increased attention modulation strength was associated with a higher proportion of hits and a lower proportion of SEs at trend level.

Examination of behavioral performance on the Dichotic Listening task suggested that participants were able to perform according to instructions. This was shown by analyzing participants’ response patterns given by the ratio of the different response types (i.e. hits, SEs, and REs). Specifically, their performance was characterized by a significantly larger share of hits compared to both types of errors. Secondly, we compared participants’ ability to detect SOA-deviants between the high- and low-predictability conditions, and found that the TDR increased in high-predictability blocks. As SOA deviants occurred less frequently in these blocks, this might have increased their saliency or novelty and therefore attracted more attention. Previous studies have shown that neural responses associated with a reorienting of attention (i.e. the reorienting negativity) increase to auditory deviant stimuli as these occur less frequently (Jankowiak and Berti 2007). Similarly, unexpected (deviant) intervals between auditory stimuli elicit neural reorienting responses (Ungan et al. 2019). Nonetheless, we interpret the effect of rhythm predictability on TDR with caution, as the number of targets varied between the two predictability conditions (i.e. less frequent SOA deviants during the high-predictability conditions). If a target was missed during the high-predictability conditions due to other factors (e.g. lapses of attention), this would impact the detection rate to a greater extent than during low-predictability conditions where targets were presented more frequently. Note that this was not an issue for the analyses of the electrophysiological data, as these focused on rhythmic sequences that did not contain SOA-deviants.

The EEG data revealed attentional modulation of power in the beta-band, in line with previous studies reporting attentional modulation of power in the alpha-beta range (for a review, see Frey et al. 2015). Contrary to earlier studies, we did not find attentional modulation of alpha-band power. While a consistent role of alpha-oscillations in functional inhibition of task-irrelevant cortical areas has been documented (for reviews, see Klimesch et al. 2007; Jensen and Mazaheri 2010), also in attentional selection (Foxe and Snyder 2011), the role of beta-oscillations is less clear (Spitzer and Haegens 2017). However, beta-suppression has been linked to cortical excitability in parietal regions (Samaha et al. 2017), as well as motor regions (for reviews, see Jensen and Mazaheri 2010; Kilavik et al. 2013). It is possible that neural alpha- and beta-band oscillations serve different roles in the attentional selection of relevant stimuli (van Ede et al. 2011). In prior studies, participants were typically asked to respond to stimulus-specific features, such as reporting certain spoken digits among distractors (Wöstmann et al. 2016), the location of tactile stimulation (van Ede et al. 2011), or orientation of visually presented Gabor patches (Heideman et al. 2018a). In the current study, participants only had to detect and respond to the occasions when the expected temporal rhythm of attended tones was violated, and did not make decisions regarding other tone features. van Ede et al. (2011) argued that alpha-power modulations may reflect anticipatory attention to both spatial position and specific features of the sensory input, while beta-band modulations primarily reflect temporal aspects of attention. Heideman et al. (2018b) reported attention modulation of lateralized beta-activity, but not alpha-activity, according to spatial expectation. Importantly, the task required speeded responses to visual stimuli at certain locations, with no decision regarding their physical features. The modulation of beta-band activity, which was also more prominent in parietal compared with visual regions, might therefore reflect the temporal anticipation and preparation for the forthcoming stimuli at the expected location. Together with these findings, our results support the assumption that the beta-band plays a specific role in the spatial–temporal aspects of selective attention (van Ede et al. 2011; Heideman et al. 2018b).

Heideman et al. (2018b) reported that the lateralization of beta-power during anticipation of spatial location was enhanced if the temporal structure of the stimuli was implicitly learned in contrast to the beta-lateralization accompanying novel stimulus sequences. Similarly, we found that the lateralization of beta-power was enhanced by rhythm predictability at source-level. In our study, the overall rhythm structure was identical throughout the entire experiment. Accordingly, the effect could not be due to having or not having learned the temporal structure. Instead, we demonstrate that modulations of the beta-band lateralization potentially reflect the implicit learning of a higher-order contextual or statistical rule (i.e. rare vs. frequent rhythm violations). This finding is in line with a growing number of studies suggesting a role of beta oscillations in temporal prediction (for a review, see Rimmele et al. 2018), and with a study showing that beta oscillations recorded directly from the human auditory cortex reflect the updating of sensory predictions (Sedley et al. 2016). Studies have reported that auditory cortical beta-activity related to temporal prediction likely results from motor-auditory cortex interactions (e.g. Fujioka et al. 2012), as well as directional coupling originating from motor and sensorimotor cortices towards auditory regions during temporal prediction (Morillon and Baillet 2017; Abbasi and Gross 2020). Our results support a role of beta-oscillations in temporal prediction by revealing that modulations are sensitive also to abstract timing rules (i.e. probability of rhythm violations) in the sensory input. This is further in line with earlier studies reporting a general role of beta-oscillations in top-down processes, and assumptions that neural activity in the beta band is crucial for long-range communication between distant cortical regions (Engel and Fries 2010; Helfrich and Knight 2016).

Crucially, we discovered that the attention modulation of beta-power synchronized more strongly to the tempo of attended compared with unattended tones. There is currently a lack of studies investigating the dynamic modulation of attention during perception of sound sequences. It has been demonstrated that mechanisms of top-down driven selective attention are inherently rhythmic and controlled by the frontoparietal attention network (Helfrich et al. 2018). In general, it has been argued that the most energy-efficient mechanism for the temporal coordination of neural processing is one that oscillates between states of increased and decreased modulation of neural activity, rather than a general enhancement of modulation over time (Buzsáki and Draguhn 2004). During recent years, a few studies have shown how attentional modulation of alpha-power synchronizes with the presentation rate of spoken words when these are to be reported afterwards (Wöstmann et al. 2016; Tune et al. 2018; Wöstmann et al. 2021). Importantly, the “to-be-reported” stimuli were presented to one ear, while distractors were presented simultaneously to the opposite ear. Contrary to these studies, where the attended and ignored stimuli were presented in synchrony, the stimuli in the current study competed in terms of both spatial location (left vs. right ear) and temporal structure (slow vs. fast rate). A central finding of this study is that beta-lateralization acts as a spatial–temporal filter aligning selectively to the relevant tempo among competing rhythmic sound streams. Hence, we demonstrate that rhythmic attentional modulation of neural activity during perception of sound sequences indeed reflects endogenous selection of the most relevant time-points, as the experimental context had simultaneous presentation of distracting stimuli at irrelevant time-points. This is further in line with a recent finding that lateralized alpha power is stronger at the onset of temporally cued stimuli within auditory rhythmic sequences (Wöstmann et al. 2021), as well as the idea that auditory selective attention dynamically adjusts to the temporal structure in behaviorally relevant auditory input streams (Large and Jones 1999).

A potential confound of the present study is that the experimental task was introduced as a motor-task before the practice sessions (i.e. instructed to tap along with the rhythm in the attended ear). Even if participants did not tap during the actual experiment, this might have affected how the task was perceived and performed. Oscillatory activity in the beta-range is known to be involved in motor-related processing (for reviews, see Engel and Fries 2010; Kilavik et al. 2013). Due to the tapping requirement during the initial practice, participants might have engaged in imaginary tapping during the actual experiment, which might explain why beta- and not alpha-band modulations were found in the present study. For example, in a recent study, Wöstmann et al. (2021) presented participants with sequences of spoken digits to both ears, with the instruction to attend to digits in one ear only, as they would have to decide afterwards which of two digits had been presented in the attended stream. Interestingly, it was found that temporal foreknowledge about which digit in the sequence was likely to be probed, led to enhanced alpha-lateralization at the onset of the temporally cued digits. This suggests that subtle differences in task designs may impact which frequency bands are modulated, possibly reflecting deployment of slightly different neurocognitive mechanisms. To determine the specific roles of the alpha- and beta-bands in attentional selection, future studies will require experiments where attention to stimulus timing and stimulus features is manipulated independently within the same context. In light of the present study, this could mean introducing experimental blocks where the task is not to respond to the time-deviances, but rather other featural changes. Furthermore, the current, as well as previous studies reporting attentional modulation of beta-power (van Ede et al. 2011; Heideman et al. 2018b), focused on the relative difference between hemispheres and not on the relative difference to a baseline, due to how the experiments were designed. Thus, it is not possible to determine to what degree the effect is driven by ipsilateral power enhancement or contralateral power suppression, according to the direction of attention. It remains an open question to what degree the beta-band is involved in the inhibition or enhancement of sensory processing in functionally irrelevant and relevant cortical areas, respectively.

Another potential limitation of the present study is how the observation of enhanced beta-lateralization with increased rhythm predictability might reflect different levels of distractibility between conditions rather than prediction per se. Specifically, the low-predictability conditions involved more frequent SOA deviants in the unattended ear. This might make the sound stream in the unattended ear capture more attention during low- compared to high-predictability conditions, resulting in less prominent attention modulation. However, in our previous study (Foldal et al. 2020) we demonstrated that the auditory N1 potential was enhanced in response to attended tones (within sequences not containing SOA deviants) during low- compared to high-predictability blocks. If the sound stream in the unattended ear was attracting more attention during low-predictability blocks, one would rather expect attention to be more distributed between the ears (i.e. divided attention), thereby resulting in attenuation of the N1 in response to attended tones (Hink et al. 1977; Parasuraman 1978). We therefore find it unlikely that the enhancement of attention modulation by rhythm predictability was driven by distraction from the unattended sound stream.

A second alternative explanation could be that enhanced beta-lateralization in the high-predictability condition is related to motor preparation, given that beta-oscillations have been linked to motor-processing (for reviews, see Engel and Fries 2010; Kilavik et al. 2013). As the amount of SOA deviants, which also represented targets, differed between the two predictability conditions, this might affect the degree of motor preparation within each condition. However, in this case one might expect enhanced modulation of beta-power in the low-predictability condition where targets are presented more frequently, which is the opposite of what was found in the present study. Accordingly, it does not seem like modulations of beta-power lateralization by temporal predictability reflects motor preparation. Nonetheless, future studies might avoid the issues related to both distractibility and potential motor confounds by making the predictability manipulation (i.e. probability of SOA deviants) independent from the behavioral task, for example by having participants detect other acoustic changes. This would permit the number of behavioral targets and distractors to be constant across experimental conditions.

Finally, we investigated the relationship between attention modulation strength at the neural level and task performance. The association between attention modulation strength and the proportions of hits and SEs did not turn out significant. However, there was a trend towards the former being associated with an increased proportion of hits and a reduced proportion of SEs, reflecting improved ability to distinguish between deviants presented to the attended and unattended ear. This is in line with previous studies reporting larger attention modulation effects (i.e. alpha-power lateralization) for correct compared with incorrect trials (i.e. Haegens et al. 2011; Boncompte et al. 2016; Wöstmann et al. 2016). As tone sequences of interest did not involve a behavioral response in the present study, there was no way to identify trials where participants might have engaged in task-irrelevant thoughts or mind wandering. A potential reason why the associations were not significant in our study might be a lack of sensitivity when examining the overall attention modulation effects and performance level, since it was not possible to directly compare accurate and inaccurate performance. Interestingly, we found additional support that the ratio of hits and SEs serves as a behavioral correlate of the attention modulation, and not simply participants’ ability to detect SOA-deviants. Specifically, there was no apparent association between their level of musical experience and amount of SEs. Instead, higher levels of musical experience were associated with an increased amount of hits and reduced amount of REs, as well as an improved TDR, more likely reflecting their ability to evaluate sound timing. Furthermore, no association was found between attention modulation strength at the neural level and level of musical experience. Hence, it appears that the balance between hits and SEs serves as a behavioral correlate of the attention modulation.

Performance variability on the Dichotic Listening task suggested that the task was demanding. Given that the study entailed a reanalysis of data initially used to investigate effects of rhythm predictability on auditory evoked responses (see Foldal et al. 2020), the left and right ear tone streams fitted together perceptually as they gave rise to a 3- vs. 4-beat meter. Participants might have been familiar with this particular rhythmic structure, making stream segregation more difficult, especially as the sounds presented to each ear were otherwise identical. A potential limitation of employing demanding tasks is that it may introduce between-subjects variability in the electrophysiological data due to trying different strategies or disengaging from the task, thereby weakening the effects at the neural level. Attention modulation of the topographical (i.e. AMI) as well as time-resolved (i.e. LI) beta-lateralization was clearly weaker at source-level. This might be due to the generally poor spatial precision of source localizing brain activity from the EEG (Brookes et al. 2008) combined with our use of a template structural MRI as well as a template for electrode positions, also known to limit the precision of the localization (Michel and Brunet 2019). Future studies planning to use templates for source-localization should consider the difficulty of the employed task. Ensuring that task-relevant and task-irrelevant sound streams remain distinguishable for all participants across the entire experimental session, can potentially reduce interindividual variability in the electrophysiological data. This could indirectly improve the quality of the source estimation.

## Conclusion

In the current study, we demonstrated that selective attention to space and time during perception of auditory rhythms (i.e. left vs. right ear sound streams with different tempos) was reflected in lateralization of beta-power according to the direction of attention. It was also shown that the lateralization of beta-power was modulated by rhythm predictability, although verification by future studies is called for as this modulation was not as clear as the other findings. Crucially, a key result of the present study was how the temporal progression of beta-lateralization, indexing attentional selection over time, aligned more strongly with the tempo of the attended- compared with the unattended competing sound stream. This supports the notion of top-down attentional control according to the *timing* of behaviorally relevant sounds. Future research should determine whether beta-oscillations play a specific role in temporal attention as opposed to feature-based attention, and to what degree the proposed neural mechanism reflects oscillatory power changes in functionally relevant and/or irrelevant cortical areas.

## Supplementary Material

SupplementaryMaterial_bhac179
