# Supplementary material for: Attentional modulation of beta-power aligns with the timing of behaviorally relevant rhythmic sounds
Source: Cereb Cortex. 2022 May 27;33(5):1876–94. doi: 10.1093/cercor/bhac179 (PMC9977362; doi:10.1093/cercor/bhac179)
Supplement: SupplementaryMaterial_bhac179 [file supplementarymaterial_bhac179.docx]

**Supplementary material**


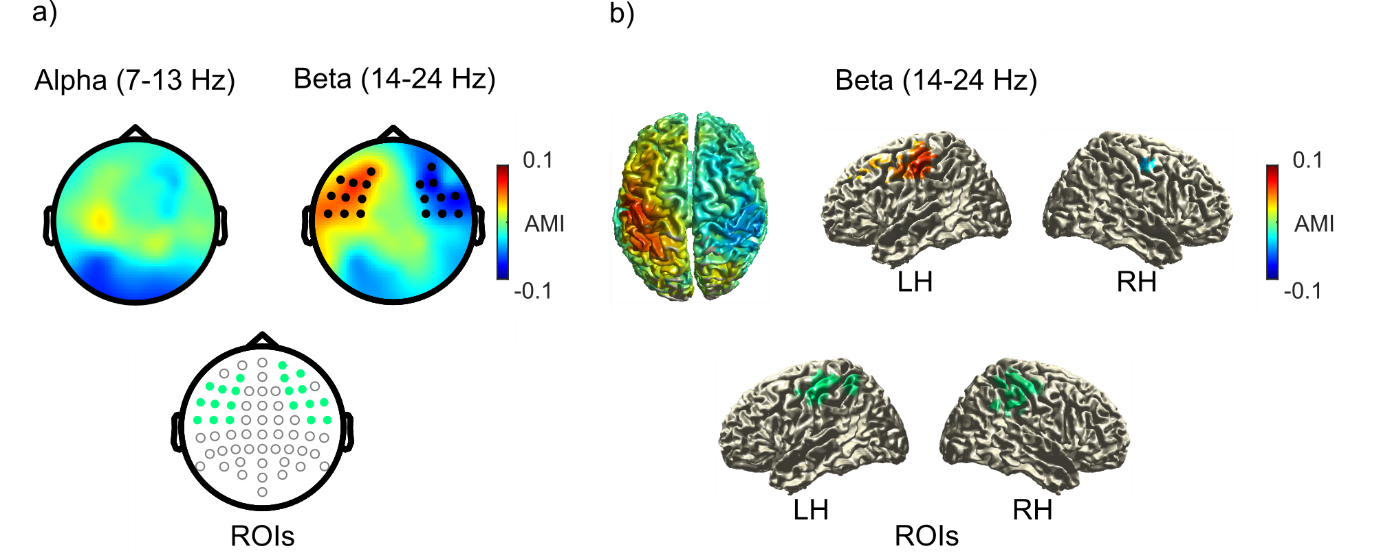


**Figure S1.** Results from the feature extraction procedure (frequency band and ROIs) performed on the pre-sequence (-200 to 0 s) time-window. (a) Topographical representation of the AMI for the alpha-band (top left) and the beta-band (top right) at scalp-level. Cluster-based permutation tests contrasting the normalized ‘attend left’ and ‘attend right’ conditions (AMI) were computed for the alpha- and beta-bands, separately. For the beta-band, the first positive (left hemisphere, *p* = .015) and the first negative (right hemisphere, *p* = .018) cluster is highlighted with black dots. No clusters were identified for the alpha-band. The resulting left and right ROIs used for analyzing data during the time-window of interest (0 to 2.2 s) are illustrated in the lower plot (green dots). (b) Topographical representation of the AMI for the beta-band at source-level (top left). A cluster-based permutation test contrasting the normalized ‘attend left’ and ‘attend right’ conditions (AMI) was computed, and the first positive (left hemisphere, *p* = .224) and the first negative (right hemisphere, *p* = .432) are illustrated in the masked left (LH) and right (RH) hemisphere surface plots (top middle and top right, respectively). The resulting left and right ROIs used for analyzing data during the time-window of interest (0 to 2.2 s) are illustrated in the lower surface plots (highlighted in green).


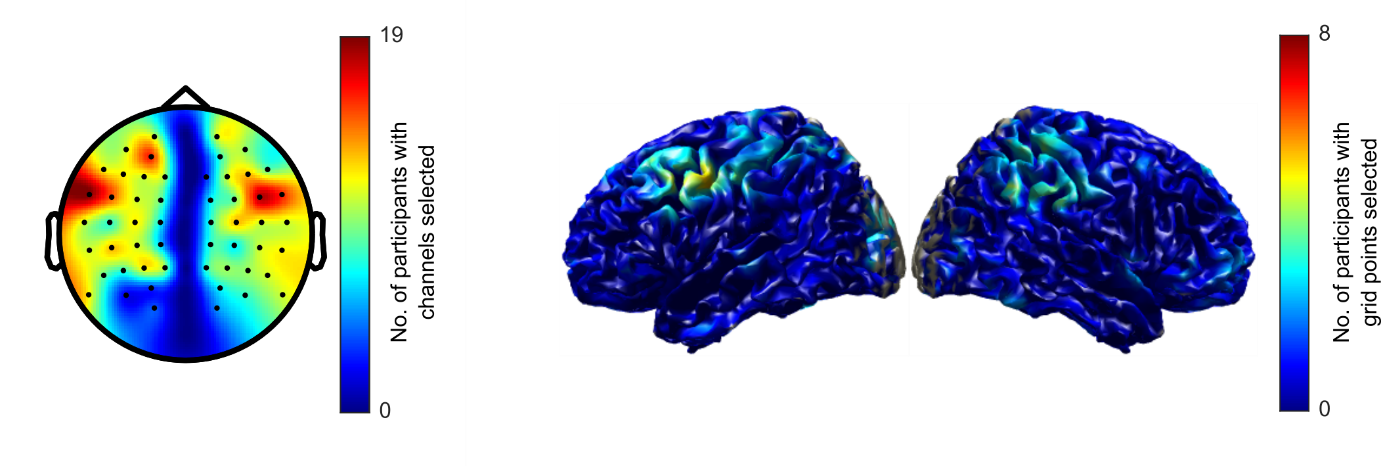


**Figure S2.** Topographic map of the selected channels (black dots, midline channels excluded) and topographic maps of the selected sources (grid points) from the left and right hemisphere across participants (total number of participants, *n* = 26). Warm colors indicate that these channels or sources were selected more frequently across participants.


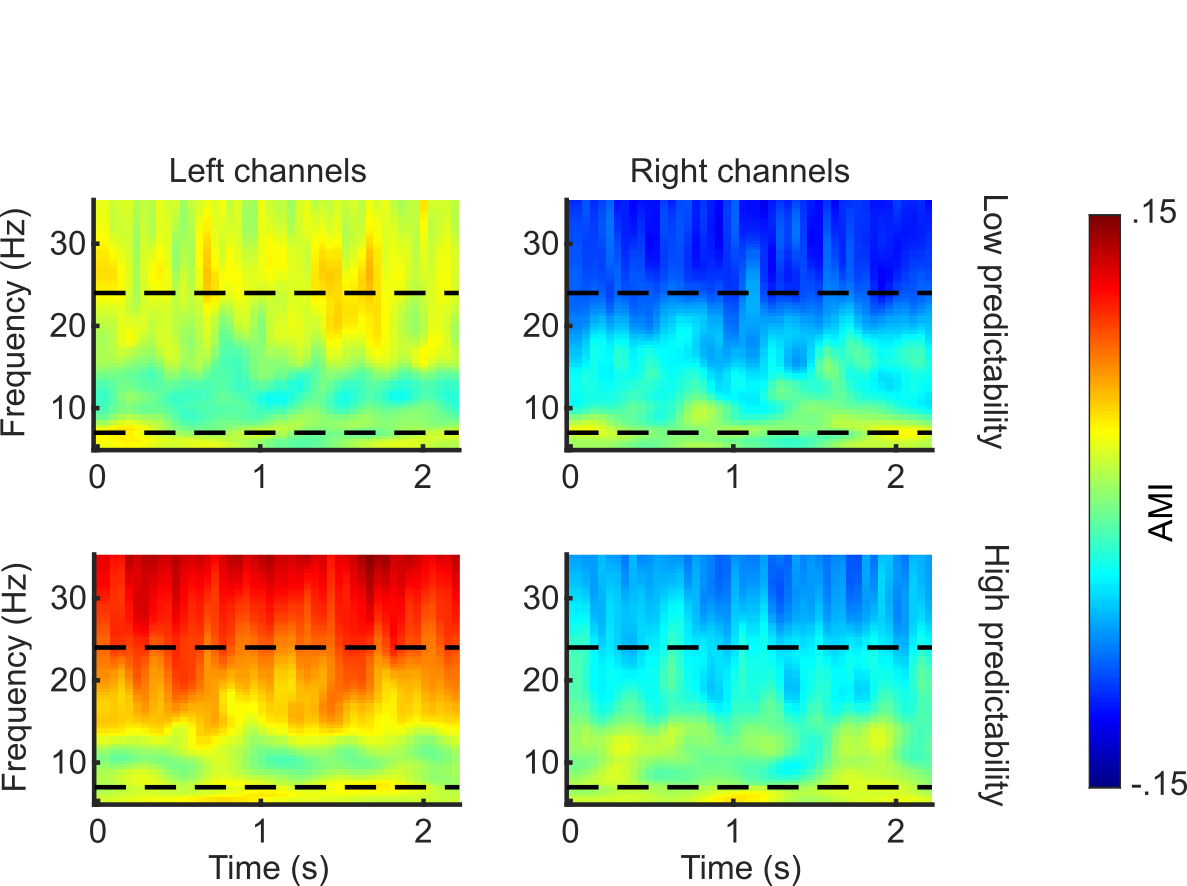


**Figure S3.** Time-frequency representation of the grand average AMI values for the left and right hemisphere channels (i.e., ROIs) for the ‘low’ (top panels) and ‘high’ (lower panels) predictability conditions. The frequency range of interest (7-24 Hz) is marked by dashed black lines.


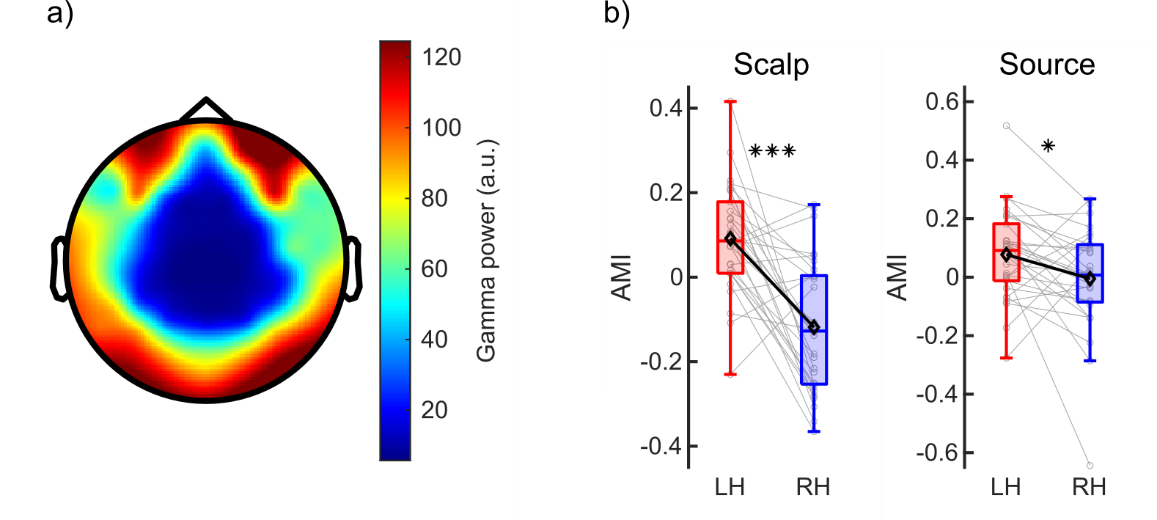


**Figure S4.** Topography of the raw gamma band (55-75 Hz) power and results from analyses of attention modulation (AMI) of gamma-power at the scalp- and source-level. (a) The topographical plot illustrates the distribution of the raw gamma power across the scalp. No baseline correction was applied to the data, and power values are presented in arbitrary units (a.u). (b) Boxplots depicting the AMI for the gamma-band at scalp-level (left) and source-level (right), illustrating the difference between AMI values over the left (LH) and right (RH) ROIs. In each boxplot, the upper and lower box edges represent the 25th and 75h percentile, respectively, and whisker length is equal to 1.5 times the interquartile range. The middle line in each box represents the median, and the mean for each condition in each plot is highlighted with a black diamond. Gray lines are drawn between pairs of individual data points. A clear difference between the left and right hemisphere was found at scalp-level, *F*(1,29) = 29.67, *p* < .001, *ω*^2^ = .345 (BF = 1.90e+5, strong evidence for a difference), while the effect was clearly reduced at source-level, *F*(1,29) = 4.88, *p* = .035, *ω*^2^ = .046 (BF = 1.34, anecdotal evidence for a difference). Note that the effect is weaker for the gamma-band data at source-level compared to what was found for the beta-band, suggesting that beta-band effects are unlikely to be driven by lateralization of gamma-power leaking into the beta band. * *p* < .05, *** *p* < .001.


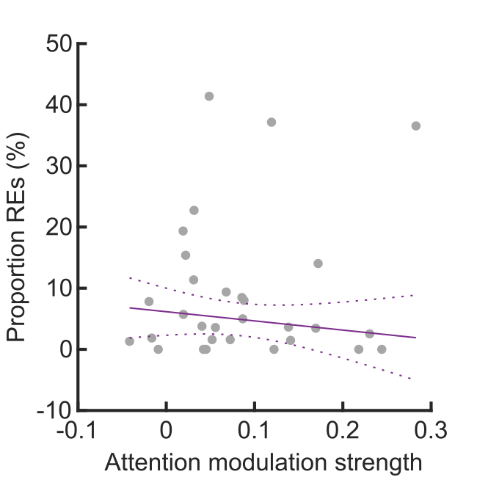


**Figure S5.** A robust-fit linear regression model of the proportion of random error responses (REs) vs. ‘attention modulation strength’. The fitted curve is given by the solid purple line, while dashed lines indicate its 95% confidence bounds. Gray dots reflect the individual data points.

**Attention modulation and effects of rhythm predictability**

The results of the repeated measures ANOVA in the reduced sample (n = 26) turned out highly similar to the full sample (n = 30). The main effect of hemisphere was significant at both scalp-level, *F*(1,25) = 26.75, *p* < .001, *ω*^2^ = .308 (BF = 5.34e+3), and source-level, *F*(1,25) = 7.62, *p* = .011, *ω*^2^ = .104 (BF = 13.43). There was no significant main effect of predictability at scalp-level, *F*(1,25) = 2.23, *p* = .148, *ω*^2^ = .030 (BF = 1.18), or at source-level, *F*(1,25) = 0.03, *p* = .861, *ω*^2^ = .000 (BF = 0.21). Finally, while the interaction of hemisphere and predictability was not significant at scalp-level, *F*(1,25) = 2.25, *p* = .146, *ω*^2^ = .007 (BF = 0.319), it remained significant at source-level, *F*(1,25) = 6.64, *p* = .016, *ω*^2^ = .050 (BF = 1.09). The enhanced effect size and more positive BF for the interaction suggested the effect to be slightly stronger in the reduced sample.


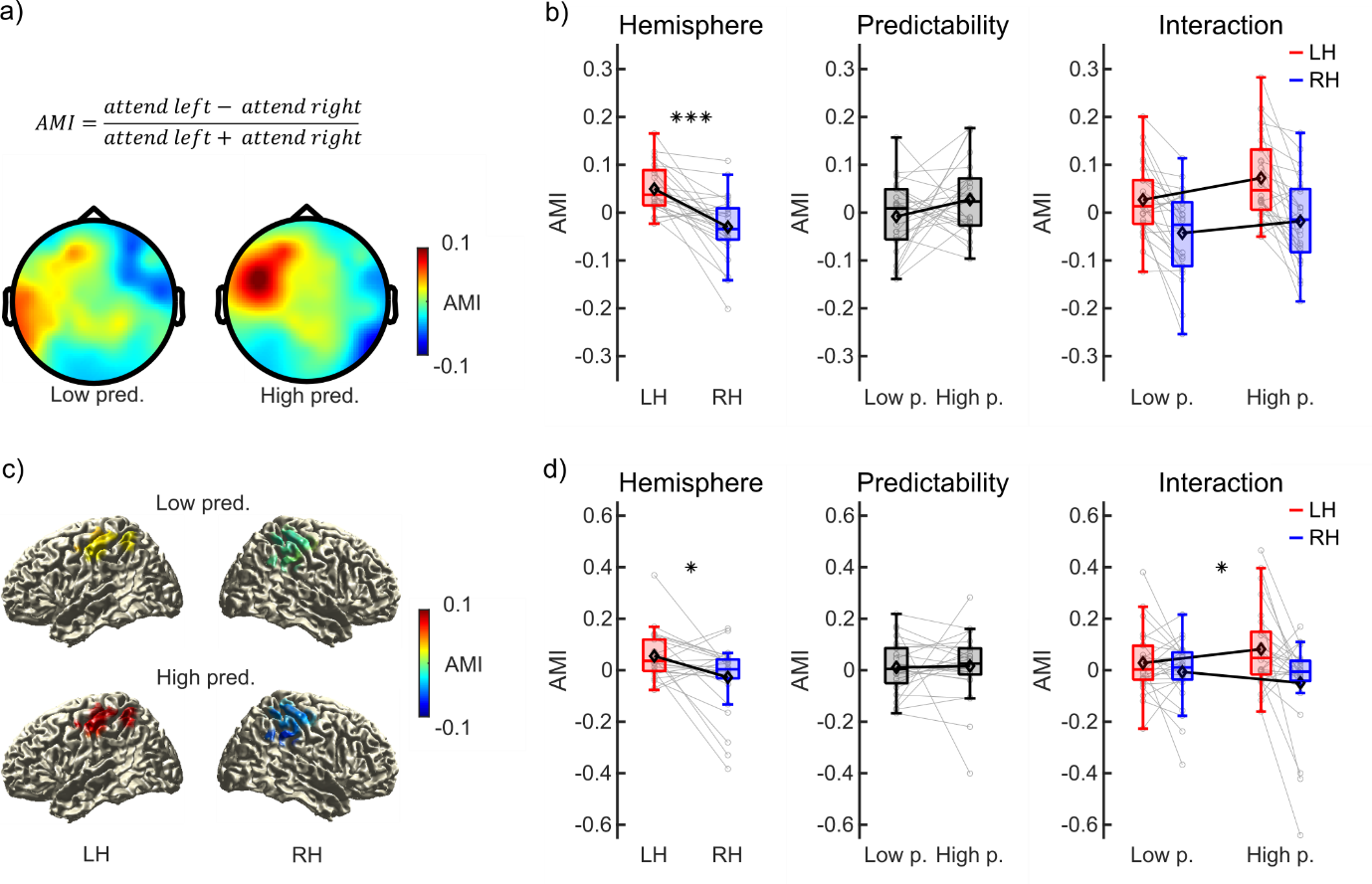


**Figure S6.** Grand average AMI topographies (scalp and source level) for the two predictability conditions and results from the rmANOVAs (n = 26). (a,c) Topographies, grand average AMI at scalp-level (a) and source-level (c), low vs. high predictability. For source-level data (c) the masks illustrate the left and right hemisphere ROIs. (b,d) Boxplots depicting the AMI for each condition at scalp-level (b) and source-level (d), illustrating the main effects of hemisphere (left), main effects of predictability (middle), and the interaction between hemisphere and predictability (right). In each boxplot, the upper and lower box edges represent the 25th and 75h percentile, respectively, and whisker length is equal to 1.5 times the interquartile range. Gray lines are drawn between pairs of individual data points. The middle line in each box represents the median. The mean for each condition in each plot (left, middle, and right) is highlighted with a black diamond. *** = *p* < .001, * = *p <* .05.


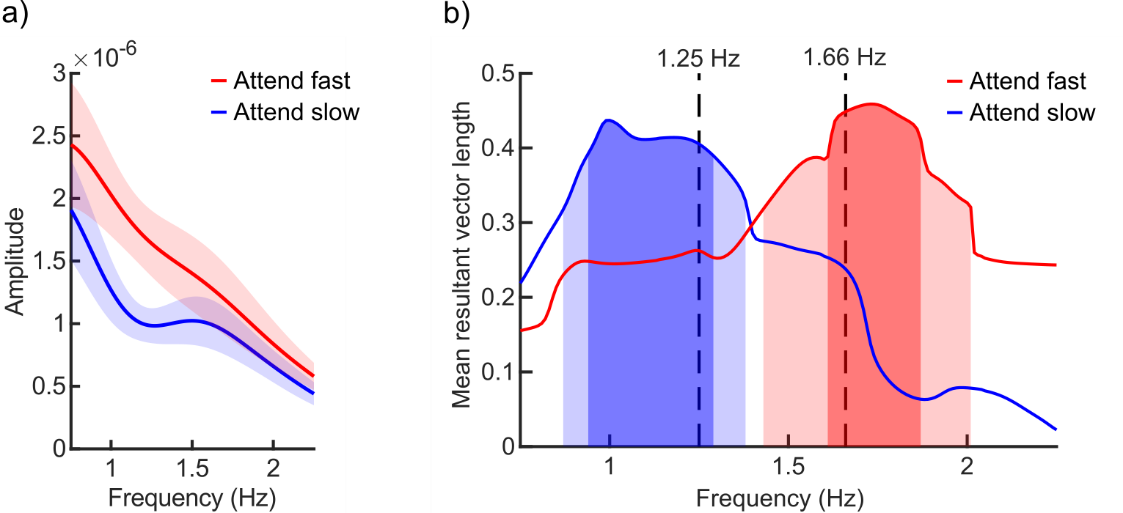


**Figure S7.** Amplitude spectra and phase-consistency (i.e., mean resultant vector length) for the ‘attend fast’ (red) and ‘attend slow’ (blue) conditions. (a) The resulting amplitude spectra from the FFTs performed on the time courses of lateralized beta power (LI) during the ‘attend fast’ and ‘attend slow’ conditions. The shaded area represents the standard error. (b) Visualization of the shift in phase consistency (i.e., mean resultant vector length) from lower to higher frequencies depending on the attended tempo (fast vs. slow). The shaded areas indicate significant *p*-values from the Rayleigh test (light shaded area, *p* < .05; dark shaded area, *p* < .01).


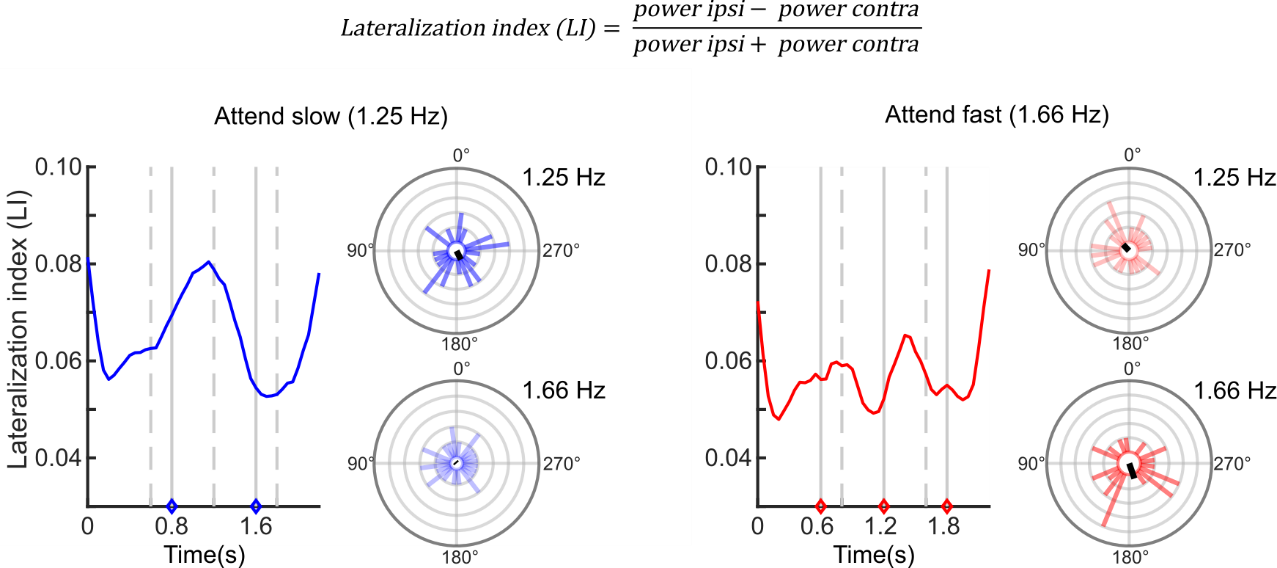


**Figure S8.** Time-courses of the lateralization index (LI) and distribution of 1.25 Hz and 1.66 Hz phase angles for source-level data during the ‘attend slow’ (left - blue color) and ‘attend fast’ (right - red color) conditions. For the time-courses, tone onsets are depicted as vertical gray lines marked as either attended (solid) or unattended (dashed) according to the attended tempo (slow vs. fast). Attended tones are additionally highlighted with diamonds on the time-axis. The distribution of both 1.25 Hz and 1.66 Hz phase angles are depicted next to each time-course, where the phases of the frequency representing the attended tempo (1.25 or 1.66 Hz) have a darker shaded color. The black line represents the mean resultant vector.
